# Phytosomes with Persimmon (*Diospyros kaki* L.) Extract: Preparation and Preliminary Demonstration of In Vivo Tolerability

**DOI:** 10.3390/pharmaceutics11060296

**Published:** 2019-06-22

**Authors:** Rosa Direito, Catarina Reis, Luís Roque, Margarida Gonçalves, Ana Sanches-Silva, Maria Manuela Gaspar, Rui Pinto, João Rocha, Bruno Sepodes, Maria Rosário Bronze, Maria Eduardo Figueira

**Affiliations:** 1 Research Institute for Medicines (iMed.ULisboa), Faculty of Pharmacy, Universidade de Lisboa, 1649-003 Lisbon, Portugal; rdireito@ff.ulisboa.pt (R.D.); mgaspar@ff.ulisboa.pt (M.M.G.); rapinto@ff.ulisboa.pt (R.P.); jrocha@ff.ulisboa.pt (J.R.); bsepodes@ff.ulisboa.pt (B.S.); mrbronze@ff.ulisboa.pt (M.R.B.); efigueira@ff.ulisboa.pt (M.E.F.); 2 Department of Biomedical Sciences, Faculty of Pharmacy, University of Alcalá, Ctra. Universidad Complutense, 28871 Alcalá de Henares, Spain; luis_roque_10@hotmail.com; 3 Departamento de Ciências e Tecnologia de Biomassa, Mechanical Engineering and Resource Sustainability Centre, Faculdade de Ciências e Tecnologia, UNL, Quinta da Torre, 2829-516 Monte da Caparica, Portugal; mmpg@fct.unl.pt; 4 National Institute for Agricultural and Veterinary Research (INIAV), Vairão, 4485-655 Vila do Conde, Portugal; anateress@gmail.com; 5 Centre for Study in Animal Science (CECA), ICETA, University of Oporto, 4099-002 Oporto, Portugal; 6 JCS, Dr. Joaquim Chaves, Laboratory of Clinical Analysis, 1495-068 Lisbon, Portugal; 7 ITQB, Estação Agronómica Nacional, Av. da República, 2780-157 Oeiras, Portugal; 8 IBET, Avenida da República, Quinta-do-Marquês, Estação Agronómica Nacional, 2780-157 Oeiras, Portugal

**Keywords:** Persimmon, *Diospyros kaki*, phenolic compounds, phytosomes, food supplement, antioxidant, encapsulation efficiency, in vivo assessment

## Abstract

Persimmon (*Diospyros kaki* L.), a fruit rich in phenolic compounds (PCs), has been considered effective in mitigating oxidative damage induced by an excess of reactive oxygen species. Due to large molecular weight and intrinsic instability in some physiological fluids, PCs’ passage through biological membranes is very limited. Carriers like phytosomes are promising systems to optimize oral absorption of encapsulated extracts. This work prepared and fully characterized phytosomes containing bioactive phenolic extracts from persimmon in terms of size, surface charge, encapsulation efficiency and stability over six months. These phytosomes were orally dosed to Wistar rats during a 15-day period. Afterwards, haematological and biochemical analyses were performed. Monodisperse phytosomes were successfully prepared, with size less than 300nm (PI < 0.3) and high encapsulation efficiency (97.4%) of PCs. In contrast to free extract, extract-loaded phytosomes had higher antioxidant activity after 6 months storage. Oral administration of extract-loaded phytosomes and free extract did not lead to lipidic profile changes and were within referenced normal ranges, as well as glycaemia levels and urine parameters. The results highlighted the potential of persimmon PCs as food supplements or pharmacological tools, suggesting a promising and safe phytosomal formulation containing bioactive agents of persimmon that could lead to health benefits.

## 1. Introduction

Recent literature reviews have shown that the majority of bioactive compounds used in modern therapeutics, about 90% of them, had natural origins, i.e., they are derived from natural products or their chemical structure was inspired by natural products [1].

In the case of dietary supplements, nutraceuticals, and functional foods based on natural products, their use is increasing in order to answer growing consumer demand. However, more evidence is necessary about their health benefits and possible risks, in order to guarantee safety and effectiveness. This study provides a contribution to a new delivery system of a *D. kaki’s* extract with the potential to exert health benefits. Several examples of phytochemical extracts of fruits and vegetables (which have strong antioxidant and anti-proliferative activity) have been described in current literature, where the combination of phytochemicals accounted for most of their total antioxidant activity. The additive and synergistic effects of phytochemicals on fruits and vegetables were responsible for their potent antioxidant activities [2].

The Ebenaceae family has more than 350 known species, one of which is the *Diospyros* genus [3]. This genus is prevalent Japan, China, and Korea, but it is in the latter two countries that Persimmon (*Diospyros kaki* L.) is traditionally used for medicinal purposes [4] for their positive effects on human health. Several different studies have reported pharmacological activities and the phytoconstituents profile of various parts of this plant [5,6,7]. Many studies showed that persimmon extract and its constituents have potent antitumor activity against human cancer cells. Even though their molecular mechanisms are not yet fully comprehended, some studies have shown that 24-hydroxyursolic acid, a triterpenoid found in persimmon, activated AMP-activated protein kinase (AMPK), inhibited cyclooxygenase (COX-2) expression in HT-29 cells, and induced cellular apoptosis by activation of poly (ADP-ribose) polymerase (PARP), caspase-3, and phosphorylation of p53 at Ser15. It also strongly induced DNA fragmentation in HT-29 cells and thereby significantly inhibited colony formation of HT-29 cells in soft agar [8]. In vitro studies also demonstrated that persimmon phenolic extract successfully impaired cell proliferation and invasion in HT-29 cells. The exposure to 250, 500, 1000 and 2000 μg/mL of persimmon phenolic extracts lowered the ability of HT-29 cells to invade the wound gap, in a dose-dependent manner and the highest concentration used (2000 μg/mL) in the study, was quite effective in inhibiting cell migration, reducing it to close to zero [9]. Further in vivo investigation in a model of colitis in mice administered with persimmon phenolic extract, showed decreased levels of expression of COX-2 and inducible nitric oxide synthase (iNOS) in the colonic tissue, these being two important mediators of intestinal inflammation, but there was no inhibition of gelatinase MMP-9 and MMP-2 activities. Given the important link between inflammation and cancer and growing evidence suggesting a role for inflammatory processes in the progression of colorectal cancer, these results highlighted the potential of persimmon polyphenols as a pharmacological tool in the treatment of patients with inflammatory bowel diseases (IBD) [9]. Accordingly, due to certain bioactive molecules like proanthocyanidins, carotenoids, tannins, flavonoids, anthocyanidin, catechin, and so forth, persimmon is considered effective in mitigating oxidative damage induced by reactive oxygen species (ROS). It should be noted that this has helped in preventing oxidation of low-density lipoproteins, in preserving the function of beta pancreatic cells, and reducing the progression of cardiovascular diseases, cancer, diabetes *mellitus*, and cell damage caused by external agents, such as the damage associated to chronic alcohol consumption [9,10,11].

However, the authors agreed that some characteristics of these phytoconstituents might limit their bioavailability, such as low solubility in aqueous media and/or low permeability of their passage through biological membranes and the intrinsic instability of these molecules in some physiological fluids [12]. However, the administration of these phytoconstituents is known to produce quantifiable pharmacodynamic effects even in the absence of pharmacokinetic determinations (e.g., bioavailability).

Nevertheless, new drug delivery strategies, such as Phytosomes^®^ (Indena, Milan, Italy), have demonstrated numerous advantages to overcome these limitations [13,14]. The encapsulation of these bioactive compounds into nanosystems has been widely accepted to be a promising strategy for the optimization of their functional or pharmacological activity after oral administration [15,16,17] and, in some cases, to improve their safety [16,18,19]. This green nanotechnology, in phytoformulations, significantly contributes to environmental sustainability through the production of nanoproducts, without causing harm to human health or the environment.

The health preventive potential of nutraceuticals is a current focus of the health-care industry given a change in paradigm within the system, shifting its principal approach from treatment to prevention. In order to guarantee compliance for the long-term use of nutraceuticals, designing oral delivery systems has become crucial for success. It is acknowledged that the use of the oral route of administration, however, is limited by the problem of low bioavailability from the gastrointestinal (GI) tract and in vivo stability of many of the nutraceuticals, for example, enzymes, certain antioxidants, vitamins, and phytoconstituents.

This study described the production of phytosomes containing a bioactive extract of *Diospyros kaki* L. based on three important requisites for their future application—high quality of a future dosage form with the potential to demonstrate efficacy and tolerability when used in humans as an add-on to the existing therapeutic options. Alongside with the preparation of phytosomes, a preliminary characterisation of tolerability after repeated dose oral administration in a rodent model was performed.

## 2. Materials and Methods

### 2.1. Materials, Solvents and Reagents

Phosphatidylcholine (48% purified from soy lecithin), 2,2-diphenyl-1-picrylhydrazyl (DPPH), Folin & Ciocalteu’s phenol reagent and quercetin 3-β-glucoside (≥90% pure) were purchased from Sigma-Aldrich, Co (St. Louis, MO, USA). Acetic acid was purchased from Panreac (Barcelona, Spain). Anhydrous sodium carbonate was obtained from VWR^®^ Prolabo^®^, VWR International. Gallic acid (98%) was acquired from Merck (Darmstadt, Germany) and methanol (99.9%) was bought from Carlo Erba Reagents (Rodano, Italy). The study made use of Milli-Q^®^ water (18.2 MΩ cm) which was attained from a Millipore—Direct Q3 UV System (Molsheim, France). Chromatography solvents are HPLC grade (Merck, Darmstadt, Germany) and the remaining reagents were analytical grade.

The materials for UHPLC-DAD: Merck^®^ gradient grade methanol; acetic acid (glacial) RPE from Carlo Erba; ultrapure water.

Standards UHPLC-DAD: A number of selected phenolic compounds that were used as standards were procured from Sigma-Aldrich^®^ (Madrid, Spain), namely: Protocatechuic acid (CAS 99-50-3); gallic acid (CAS 149-91-7); chlorogenic acid (CAS 329-97-9),;caffeic acid (CAS 331-39-5); epicatechin (CAS 490-46-0); ferulic Acid (CAS 1135-24-6); *p*-coumaric (CAS 7400-08-0); catechin (CAS 88191-48-4); and fisetin (CAS 345909-34-4); all from Sigma-Aldrich. The purity of the standards was always greater than 95%.

Sticks URIT 10V were purchased from Quilaban LDA (Quilaban LDA, Sintra, Portugal).

Ketamine (Imalgene^®^ 1000) was purchased from Merial (Lisbon, Portugal), and xylazine (Rompun^®^ 2%) was purchased from Bayer (Lisbon, Portugal).

### 2.2. Equipments

The Vötsch Industrietechnik heating chamber, VC2033 mit TC-Steuerung; Centrifuge 5804 R from Eppendorf (Hamburg, Germany); Rotavapor R-210 from Buchi (Meierseggstrasse, Switzerland; magnetic stirrer (HTS 1003, LMS, Tokyo, Japan); UV-vis spectrophotometer, Hitachi L-2000 (Hitachi High Technology, Tokyo, Japan); Millipore^®^ water system (18.2 Ω cm at 25 °C) was purchased from Millipore—Direct Q3 UV System equipment (Molsheim, France); Delsa Nano C (Coulter, CA, USA); scanning electron microscopy (SEM 5200LV, JEOL, Tokyo, Japan) to evaluate morphology. A HPLC-DAD system (SpectraSystem, Thermo, Darmstadt, Germany) equipped with a binary gradient pump (SpectraSystem P2000, Thermo, Darmstadt, Germany), automatic sampler (SpectraSystem AS1000, Thermo, Darmstadt, Germany), diode array detector (SpectraSystem UV6000LP, Thermo, Darmstadt, Germany), UV controller (SpectraSystem SN4000, Thermo, Darmstadt, Germany) and software Xcalibur™ 2.0.6 (Thermo Fisher Scientific Corporation, Waltham, MA, USA) was used as well as an UHPLC Acquity^TM^ (Waters, Milford MA, USA) equipped with a binary pump, an auto-sampler binary solvent manager, a column thermostatting system and a diode array detector (DAD).

### 2.3. Methods

#### 2.3.1. Vegetal Extract Preparation

The fruit used in this study, Persimmon (*Diospyros kaki* L.), was acquired from a cultivar grown in Portuguese territory (Setúbal region). The extract of persimmon fruit was prepared via the union of a number of methods described [20,21] albeit with some adjustments. Approximately 360 g ± 0.1 g of fresh persimmon fruit (FW) was extracted without calyx, using acetone:water (80:20, *v*/*v*) and it was then mechanically homogenized for 10 min (at room temperature and protected from light). The extracted sample was then centrifuged at 8603× *g* for 10 min at room temperature (Eppendorf centrifuge 5804 R (Hamburg, Germany). The supernatant was then collected, and the pellet was extracted three more times using this same procedure. The supernatants were collected together and filtered, the sample was evaporated to almost dryness (35 °C, with slow, adjustable rotation), and finally, the extract was made up of Milli-Q^®^ water (240 mL) with at least 1.5 g FW/mL to encapsulate. The extract obtained was subsequently subdivided into aliquots and it was stored in falcon tubes at −20 °C, for further analysis.

#### 2.3.2. Quantification of the Total Polyphenolic Content

The procedure to quantify total phenolic content determination was based on a previous work [22]. An aliquot of the sample with 100 µL was added to 200 µL of Folin-Ciocalteau reagent (diluted in water at 1:10, *v*/*v*) where after 3 min, 1 mL of sodium carbonate (15%, *w*/*v*) and 2 mL of water were added. After incubating at room temperature for one hour, the absorbance was measured in the UV-Visible spectrophotometer at 765 nm (Hitachi L-2000, Hitachi High Technology, Tokyo, Japan) against a blank, i.e., a mixture of water and reagents.

A calibration curve of gallic acid was performed in a concentration range from 2 to 500 mg/L. The results were expressed in milligrams of gallic acid equivalents (mg of GAE) per 100 g of fresh fruit and per millilitre of extract. The determinations were made in triplicate and results were expressed as the means ± SD.

#### 2.3.3. Quantification of *D. kaki* Phenolic Compounds in Extract and Non-Encapsulated PCs by Liquid Chromatography 

An Ultra High Performance Liquid Chromatographic (UPLC^®^, Acquity^TM^, Waters, Milford, MA, USA) method coupled with a diode array detector (UHPLC-DAD) was developed for simultaneous quantification of the main phenolic compounds of the persimmon extract. The pre-column was an Acquity™ UPLC^®^ BEH C18 (2.1 mm × 5 mm, 1.7 μm particle size). The column was an Acquity™ UPLC^®^ BEH C18 RP 18 (2.1 mm × 50 mm, particle size 1.7 μm). The mobile phase was a gradient of water with 0.1% acetic acid (*v*/*v*) (solvent A) and acetonitrile with 0.1% (*v*/*v*) acetic acid (solvent B). The mobile phase gradient was: 0 min, 10% of solvent B; 2.5 min 15% of solvent B; 10 min, 30% of solvent B; 10.5 min, 30% of solvent B; 12 min 10% of solvent B.

The temperature of the analytical column was 20 °C and the samples were kept at 5 °C until analysed. The injection volume was 10 μL and the flow of the mobile phase was 0.2 mL/min. In order to correctly identify each compound, specificity was evaluated by comparing the absorption spectra of the chromatographic peaks obtained from the sample with the absorption spectra of analytical standards [23,24]. The quantification of the compounds was performed based on the peak area at a characteristic maximum absorption spectrum (λ_max_) and the corresponding retention time (R_t_) of each phenolic compound. The data was acquired using the software Empower™ version 2.0 (Waters, Milford, MA, USA). The analyses were performed in triplicate.

The chromatographic separation by high-performance liquid chromatography (HPLC) was performed on a reverse phase C_18_ column (Thermo Scientific) with a particle diameter of 5 μm and a length of 15 cm. The acquisition of chromatograms and UV-Vis spectra was performed using the software Xcalibur™ 2.0.6 (Thermo Fisher Scientific Corporation, Waltham, MA, USA), scanning between 190 and 700 nm, with a range of 1 nm and also at the specific lengths of 280 and 360 nm. The injection volume was 20 µL. The mobile phase consisted of Milli-Q^®^ water–phosphoric acid (99.9: 0.1, *v*/*v*) as eluent A, and Milli-Q^®^ water–acetonitrile–phosphoric acid (59.9:40.0:0.1, *v*/*v*/*v*) as eluent B. The mobile phase gradient was: From 0-15 min was from 0% to 20% of solvent B; 10 min with 20% solvent B; 25–70 min, from 20% to 70% solvent B; 70–75 min, with 70% of solvent B; 75–85 min from 70% to 100% solvent B; 85–110 min, with 100% solvent B; 111–120 min 100% of solvent A. The flow rate was 0.7 mL/min. Xcalibur™ software version 2.0.6 (Thermo Fisher Scientific Corporation, Waltham, MA, USA) was used to acquire and process the data. The identification of compounds was done by comparing the retention time (R_t_), spectra and spiking samples with pure standard solutions, whenever available, or comparing with data from the literature.

An aliquot of the concentrated extract was diluted (1:7) in Milli-Q water to a concentration equal to the one present in the formulation and was analysed using the method and assay conditions described above. The compounds present in the supernatant of the formulation were identified by comparing and overlapping of the chromatograms obtained for the extract.

#### 2.3.4. Preparation of Standard Stock Solutions and Work Solutions

From each compound, stock solutions at 5 mg/mL were prepared, except for protocatechuic acid with 2 mg/mL. The dilutions (1:100) of those stock solutions were performed with acidified 50% methanol (0.1% acetic acid) and filtered with a 0.2 μm filter. Several standard concentrations were then prepared: Gallic acid (50 μg/mL); chlorogenic acid (125 μg/mL); caffeic acid (50 μg/mL); epicatechin (125 μg/mL); *p*-coumaric acid (50 μg/mL); ferulic acid (75 μg/mL); (+)-catechin (150 μg/mL); fisetin (50 μg/mL) and protocatechuic acid (50 μg/mL). To this mixture, 6.8 mL of methanol was added and the volume of the volumetric flask was made up to 20 mL with ultrapure water with 0.2% acetic acid. Subsequently, approximately 2 mL of the solution was filtered into vial with 0.2 μm filter and individually injected into the UHPLC-DAD.

The order of elution of phenolic compounds was observed in the chromatogram corresponding to the mix solution. For each phenolic compound, the characteristic maximum absorption spectrum (λ_max_) and the retention time (R_t_) was determined [25].

#### 2.3.5. Sample

The persimmon extract solution was prepared with 1.5 mL of mature persimmon extract in a 10 mL volumetric flask and the volume of each flask was completed with acidified 50% methanol (0.1% acetic acid). Approximately, 2 mL of this sample solution was filtered for vials with 0.2 μm filters, for further analysis in UHPLC. The results were expressed in mg/100 g of fresh fruit.

#### 2.3.6. Phytosomes Preparation

Phytosomes were prepared by adding extract to phosphatidylcholine dissolved in 20 mL of ethanol (1:1 or 1:2, molar ratio). This mixture was heated to 25 °C with a rotation of 300 rpm (HTS 1003, LMS, Tokyo, Japan) for 2 h. Thereafter, 40 mL of 2% acetic acid solution was added, and the mixture remained for 24 h in the same conditions previously described by Matias et al. 2015 [26].

#### 2.3.7. Physical Characterization of Phytosomes 

The phytosomes were diluted (1:10) with distilled water and analysed on a Delsa Nano C (Coulter, CA, USA). The mean particle size, polydispersity index (PI) and zeta potential (ζP) were evaluated at room temperature using detection angle of 165° (except for zeta potential at 90°) for samples of the 1:1 and 1:2 formulation after 0, 3 and 6 months of preparation.

#### 2.3.8. Determination of Encapsulation Efficiency (EE)

The efficiency of encapsulation (EE, %) of the extract in the phytosomes was determined by evaluating the fraction of the non-encapsulated extract [27]. After centrifugation, the supernatant was collected and analysed on HPLC-DAD. Briefly, 2 mL aliquot of the phytosomal formulation was added to 2 mL oil and homogenized. Then, it was centrifuged at 15,000× *g* (Eppendorf centrifuge 5804 R (Hamburg, Germany) for 30 min at 4 °C. The lower phase was separated and filtered with a 0.22 μm filter (VWR^TM^, Radnor, PA, USA) and injected for HPLC chromatographic analysis. A blank was prepared in the same manner but with 2 mL water and 2 mL oil. The encapsulated extract (%) was calculated by the difference between the total persimmon extract area (corresponding to the extract added to the formulation) and the total chromatogram area of the supernatant (corresponding to non-encapsulated phenolic compounds) [26,28,29,30]. Applying Equation (1), the encapsulation efficiency (EE, %) of the extract in the phytosomes was determined at 280 and 360 nm. Independent injections into the HPLC column were performed and analysed.

(1) $$\mathrm{EE}\;\left(\%\right)=\frac{W\left(\mathrm{added}\;\mathrm{extract}\right)-W\left(\mathrm{free}\;\mathrm{extract}\;\mathrm{in}\;\mathrm{supernatant}\right)}{W\left(\mathrm{added}\;\mathrm{extract}\right)}\times 100$$

The total phenolic compounds in the supernatant were also calculated by the spectrophotometric (765 nm) quantification of total phenolic compounds by the Folin-Ciocalteu technique. The same calculation was made for the extract sample and the encapsulation efficiency (EE, %) of the extract in the phytosomes was also calculated according to Equation (1). All measures were performed in triplicate.

#### 2.3.9. Stability Test over the Time

The objective of the stability study was to evaluate the stability based on the test of at least three batches of the formulation following international guidelines [31]. Stability studies should include testing of those attributes of the bioactive compounds that are susceptible to change during storage and are likely to influence quality, safety, and/or efficacy [31,32].

Parameters like particle size, PI and zeta potential were accessed over time.

#### 2.3.10. Storage Conditions

In general, a consumer product should be evaluated under storage conditions (with appropriate tolerances) and, if applicable, its sensitivity to moisture or potential for loss of solvent. The storage conditions and duration of the chosen studies should be sufficient to cover the storage, transport, shipping and subsequent use [31].

In an intermediate stability study, the minimum time period covered by data at submission was 6 months and the storage conditions generally were 30 ± 2 °C/65% RH (relative humidity) ± 5% RH [32].

The accelerated storage conditions of the study were: 40 ± 2 °C/75% RH ± 5% RH; 25 ± 2 °C / 60% RH ± 5% RH and 4 ± 2 °C, over a period of 6 months. In the accelerated storage condition, a minimum of three points in time, including the start and end time points (for example, 0, 3 and 6 months), of a 6-month period study was recommended [32]. 

#### 2.3.11. Closure System and Container

Stability studies were conducted by storing the concentrated polyphenolic extract in a container and closure system, which was the same as the proposed packaging for storage and distribution [31].

The two formulations (1:1 and 1:2, molar ratio) with 30 mL were each stored for 6 months in glass amber type II colour bottles and sheltered from light at room temperature (~25 °C) and 60% RH, in the refrigerator at 4 °C and 55% RH and at 40 °C in a heating chamber (Vötsch Industrietechnik, VC2033 mit TC-Steuerung, Oldenburg, Germany) with 75% humidity. At the same time, aliquots (7 mL) of the concentrated extract as well as quercetin standard solutions (10 mL) were also placed under the same conditions as the above-mentioned formulations.

#### 2.3.12. Determination of the Antioxidant Activity

The potential for elimination of free radicals by the extract and by the phytosomal formulation (1:1 and 1:2) was determined according to the previous procedure [33]. In brief, aliquots of 10 μL of each sample were mixed with 990 μL of DPPH solution (0.002% in 70% ethanol). The reaction mixture was incubated at room temperature for 30 min in the dark. The positive control was quercetin at 10 mgmL^−1^ in water. As the absorbance control (i.e., at 100%), a sample containing 10 μL water and 990 μL DPPH was prepared. The free radical scavenging potential of the 1:1 and 2:1 phytosomal formulation and free extract samples were expressed as the disappearance of the initial purple colour. The absorbance was measured at 517 nm against a blank sample of 70% ethanol using a UV-Visible Spectrophotometer (Hitachi L-2000 from Hitachi High Technology, Tokyo, Japan). Each sample was tested in triplicate (*n* = 3; mean ± SD). The sequestration capacity of DPPH was calculated using the following formula (2):

(2) $$\mathrm{Scavenging}\;\mathrm{activity}\;\left(\%\right)=\;\frac{\mathrm{Absorbance}\;\mathrm{control}-\mathrm{Absorbance}\;\mathrm{sample}}{\mathrm{Absorbance}\;\mathrm{control}}\times 100$$

#### 2.3.13. In Vivo Safety Assessment

Four-month-old female Wistar rats (250–330 g), obtained from Charles River (Barcelona, Spain) were housed in a 12–12 h light-dark cycle with a constant temperature environment of around 22 °C and a relative humidity of 55%. Animals were allowed free access to food and water during acclimatization. All animal experiments were conducted according to the animal welfare organ of the Faculty of Pharmacy, Universidade de Lisboa, approved by the competent national authority *Direção-Geral de Alimentação e Veterinária* (DGAV) and in accordance with the EU Directive (2010/63/UE) and Portuguese laws (DR 113/2013, 2880/2015 and 260/2016).

Animals were randomly allocated into three experimental groups: The sham group which were dosed with distilled water (*n* = 3); test group with animals orally dosed with extract-loaded phytosomes (15 mg/kg) (*n* = 5); and the free extract group (*n* = 5) where the same dose of the extract was applied. Oral administrations were performed daily, during the 15 days of the protocol, by gastric gavage. During the 15-day experiment, weight and mortality of all animal groups were monitored. The clinical signs of each rat were examined for ataxia, dehydration, dyspnoea, hypothermia, tachypnoea, lack of movement, sustained rapid movement around the cage, seizure, hunched posture and ruffled fur. A scale of 0 to 3 was used by an investigator blinded to treatment groups, where 0 was used for no indication, 1 for minimal indication, 2 for critical indication, and 3 for extreme indication of clinical problem(s).

Glycaemia was measured three times during the 15-day assay, at day 0, at the end of the first week of administration, and at day 15 of the protocol. All measures were done in triplicate in each of the three times of the protocol.

For the analysis of urine parameters, urine of all animal groups was collected on days 0, 7 and 15 and urine density, pH, bilirubin, urobilinogen, blood, glycosuria, proteinuria and ketonic bodies, leucocytes and nitrites were analysed using Sticks URIT 10V (Quilaban LDA, Sintra, Portugal). All measures were done in triplicate in each of the three times of the protocol.

After 15 days, animals were anaesthetised, blood and urine were collected for analysis and then sacrificed. 

The evaluation of biochemical and haematological parameters was performed on the blood and serum of all animals. The blood was taken via cardiac puncture, where whole blood was collected into two tubes containing ethylenediaminetetraacetic acid (EDTA) as an anticoagulant. For haematological analysis, the parameters examined were: Mean corpuscular volumes (MCV); mean corpuscular haemoglobin (MCH); mean corpuscular haemoglobin concentrations (MCHC); neutrophils (NEU, %); basophils (BAS, %); eosinophils (EO, %); monocytes (MO, %) and lymphocytes (LYM, %); erythrocyte counts (RBC); leukocyte counts (WBC); haematocrit, haemoglobin (Hb); and platelets (Coulter MaxM, Beckman Coulter, High Wycombe, UK). Another part of the blood was collected into tubes without EDTA and was centrifuged after coagulation (2500× *g* during 10 min). The serum was analysed by an automated clinical chemistry analyser (ADVIA^®^1200, Synchron CX4, Beckman Coulter, Bucks, UK). Biochemical parameters analysed were cholesterol (CHOL), low density lipoprotein (LDL), high density lipoprotein (HDL), alanine aminotransferase (ALT), alkaline phosphatase (ALP), aspartate aminotransferase (AST), triglycerides (TRIG), urea (UR), total proteins (T-PROT), and creatinine (CR). The results of each tested parameter have been presented for each group of rats as the mean ± SD.

#### 2.3.14. Statistical Analysis 

The results were expressed as the means ± standard deviations (SD). An analysis of variance with ANOVA test was applied. For multiple comparison group tests, Tukey’s or Dunnett´s multi-comparison test was applied, using Graph Prisma Version 6.01 (GraphPad Software, San Diego, CA, USA). The differences were considered statistically significant when *p* ˂ 0.05.

## 3. Results

### 3.1. Quantification of the Total Phenolic Content of D. kaki 

The Folin-Ciocalteau method was performed using gallic acid as standard and a range from 2–500 mg/L was considered linear (*R*^2^ = 0.9868) and was used to determine the concentration of total phenolic compounds in the extract prepared for this work. The results are presented in Table 1.

### 3.2. Quantification of the Phenolic Compounds in Extract of D. kaki by UHPLC-DAD

The concentrations of the phenolic compounds (gallic acid, protocatechuic acid, chlorogenic acid, catechin, caffeic acid, epicatechin, *p*-coumaric acid, ferulic acid and fisetin) were determined by the UHPLC-DAD methodology. The retention time (R_t_) and the characteristic maximum absorption wavelength (λ_max_) of each phenolic compound were determined. This method allowed for the detection and quantification of the phenolic compounds at low concentrations (LOD < 0.125 μg/mL, except for catechin which presented a LOD of 0.3 µg/mL) as described in Table 2. 

Calibration curves were obtained for each of the studied compounds. The areas of the peaks were measured at the wavelength (λ) characteristic of each phenolic compound in the extract. The determination coefficients were obtained according to the ICH guidelines [23,24] and all values were higher than 0.999, thus it was evidenced that the method was linear for all PCs in the working ranges used [25]. The concentration of each phenolic compound present in the extract was determined (Table 3).

In this study, the gallic acid was the most abundant phenolic compound in the extract. The observed value was similar to the one reported by Pu et al. (2013) which found a concentration of 2.789 mg/100 g fresh weight (FW) in the variety *D. kaki var. silvestris* M [34]. However, Veberic and colleagues reported 2.43 ± 0.215 mg/100 g FW [35]. The levels of the three hydroxycinnamic acids (chlorogenic acid, caffeic acid and ferulic acid) were below 0.1 mg/100 g FW, except for the chlorogenic acid content, as described by Pu et al. [34]. The chlorogenic acid concentration in this study was 0.171 mg/100 g FW *D. kaki* extract. It is possible to find published results of 0.274 mg/100 g FW in the variety of *D. kaki var. silvestris* M and 0.145 mg/100 g FW in the variety of *D. kaki* cv. Xingyangshuishi [34]. The same authors did not find *p*-coumaric acid in 5 of the 6 genotypes analysed. The only genotype where *p*-coumaric acid was detected was in *D. kaki var. silvestris* M, with a value of 0.048 ± 0.004 mg/100 g FW [34], approximately twice less the concentration found in this study 0.097 ± 0.004 mg/100 g FW.

Comparing with hydroxycinnamic acids [34,35], the amount of hydroxybenzoic acids was higher in the tested extract but the (+)-catechin and (-)-epicatechin contents were lower than previous values reported [36,37]. The same was observed with flavone fisetin [38]. However, it should be taken into consideration that the different stages of maturation and the different cultivar which might lead to a wide variability [6].

### 3.3. Physical Characterization of Phytosomes

The characterization parameters of the phytosomal formulation by dynamic laser scattering (DLS) technique after 0, 3 and 6 months of storage are described in Table 4.

**Figure 1:**
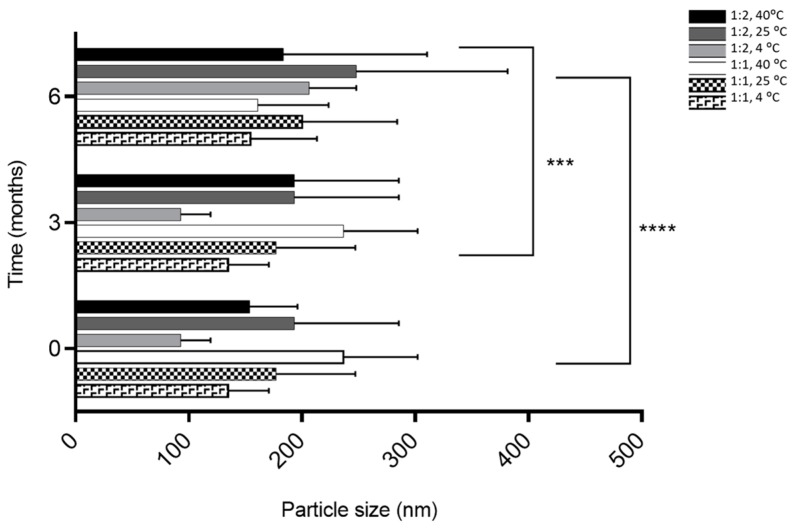
The influence of storage conditions of T and RH in phytosomal formulation size. **** *p* ˂ 0.0001—0 months vs. 6 months, *** *p* ˂ 0.05—3 months vs. 6 months.

**Figure 2:**
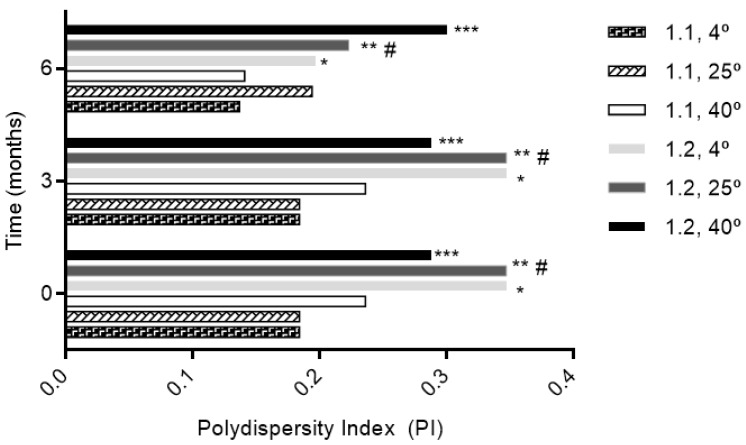
The influence of storage conditions of T and RH in PI of phytosomal formulation. * *p* ˂ 0.05 vs. 1:1, 4 °C; ** *p* ˂ 0.05 vs. 1:1, 4 °C; *** *p* ˂ 0.05 vs. 1:1, 4 °C; # *p* ˂ 0.05 vs. 1:1, 25 °C.

The size was similar regardless of the molar ratio with a size less than 300 nm (Figure 1). Phytosomes were monodispersed (PI < 0.3) regardless of the storage temperature for the 1:1 formulation, excluding the 1:2 formulation at 25° and 4 °C during the first 3 months (Figure 2). The high lipid composition in the formulation (1:2) increased the tendency for the formation of agglomerates (PI = 0.347, for 1:2 formulation at 25° and 4 °C), according to a study by Surini and colleagues [39]. 

The general values of the zeta potential of these phytosomes were negative (Table 4). The zeta potential was between −41.74 and −27.62 mV. The negative charge produced from the ethanol used in phytosome preparation may prevent the aggregation due to electrostatic repulsion and resistance forces [39]. The Surface charge expressed as zeta potential is an important physicochemical parameter that influences the stability of nanosuspensions which may also influence biodistribution, pharmacokinetics, and cellular affinity and drug internalization [40]. When compared to a positive one, the literature stated that negative zeta potential has been generally associated to higher biocompatibility [41,42].

### 3.4. Determination of Encapsulation Efficiency (EE)

#### 3.4.1. EE Assessed by High-Performance Liquid Chromatography (HPLC) Analysis

**Figure 3:**
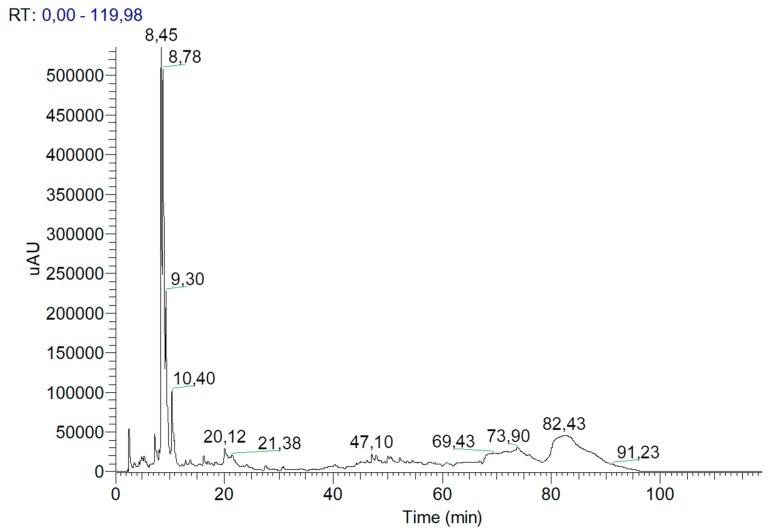
The chromatographic profile at 280 nm of the extract present in the formulation analysed by HPLC.

The chromatographic profile at 280 nm of the extract in the formulation is presented in Figure 3.

The most abundant compounds identified at this wavelength were gallic acid and catechin. At 360 nm, the chromatographic profile showed that compounds like kaempferol and quercetin could be present in glycosylated forms. These results are in accordance with the literature [43,44,45].

Considering the total peak area at 280 nm, it was concluded that the phytosomes were able to encapsulate 97.4% of the total phenolic content in the extract, encapsulating 99.3% of gallic acid present in the extract. 

These results showed a high capability of encapsulation of the phytosomes for the extract. 

#### 3.4.2. EE Assessed by Spectrophotometric Analysis

The EE of the extract by phytosomes was further verified by Folin-Ciocalteau spectrophotometric methodology (Table 1). The concentration of total phenolics was 1112 ± 34.6 mg GAE/L, and in the supernatant was 70.9 ± 4.9 mg GAE/L. Based on this methodology, the value of EE was 93.6%, again more than 90%. This value confirmed the high EE already determined. 

The differences found between the determination of total phenolic compounds by spectrophotometric Folin-Ciocalteau method and HPLC, may be due to the fact that the Folin-Ciocalteau technique is not specific, also reacting with non-phenolic compounds [46,47,48] as sugars. As PCs are the most abundant antioxidants in most plants, this method is still the most used for the determination of total phenolic content [48]. 

### 3.5. Determination of Antioxidant Activity (DPPH)

**Figure 4:**
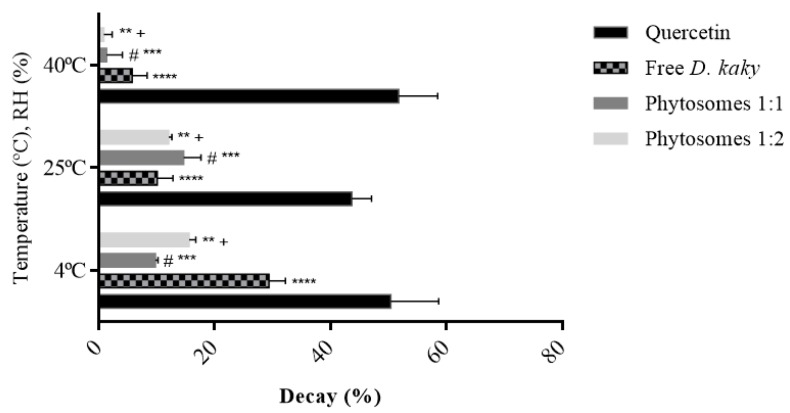
The percentage of decay of antioxidant activity of the free extract, phytosomes (molar ratio 1:2 and 1:1) and quercetin, in the conditions of temperature and humidity established (%DPPHt_0_ –%DPPHt_6_). **** *p* ˂ 0.0001 vs. quercetin, # *p* ˂ 0.0001 vs. quercetin, ** *p* ˂ 0.0001 vs. quercetin, *** *p* ˂ 0.0001 vs. free extract, + *p* ˂ 0.0001 vs. free extract.

These results, presented in Figure 4, demonstrated that these phytosomes maintained the behaviour of the extract, amplifying its antioxidant potential over time. In contrast to the free extract, extract-loaded phytosomes had higher antioxidant activity over 6 months. Specifically, at 40 °C, the decay of the antioxidant activity demonstrated by the 1:1 and 1:2 loaded phytosomes were respectively, four and six times lower than that demonstrated by the free extract under the same conditions. The quercetin standard had a decay of 51.8%, approximately nine times higher than that of the free extract. When compared to loaded phytosomes, the quercetin standard declined 38 times more than the 1:1 formulation and 57 times more than the 1:2 formulation.

At 25 °C, the decay of antioxidant activity of the extract and the phytosomes followed the same tendency, approximately between 10% and 15%. On the other hand, the decay of antioxidant activity for the quercetin standard was 43.73%, which is four times higher than that observed in the free extract, three times more than the loaded phytosomes with the highest decay (1:1), and approximately four times higher than 1:2 phytosomes under the same storage conditions. At 4 °C, the quercetin standard exhibited a decay of antioxidant activity of 50.36%. The free extract under the same conditions had a decay of this activity of 29.44% and the phytosomes between approximately 10% and 16%.

Overall, it was found that phytosomes have a smaller loss of activity over the course of 6 months, particularly at 40 °C, suggesting that these carriers maintained the behaviour of the extract and amplifyied its antioxidant activity over the study time in the storage conditions of temperature (T) and relative humidity (RH). 

This encapsulation process is still a very useful process utilized in the incorporation of active ingredients aiming, among other objectives, to obtain a modified release of pharmaceutical compounds or protect them from harsh atmospheric agents (moisture, light, heat and/or oxidation). One similar example was also observed with extracts obtained from *Plectranthus* [27]. After encapsulation, the major compounds like chlorogenic acid (CA) and rosmarinic acid (RA) were encapsulated in calcium alginate beads and they were stable over time and against UV in contrast to free extracts (or non-encapsulated). 

### 3.6. Evaluation of In Vivo Experiment

#### 3.6.1. Changes in Weight and Mortality

**Figure 5:**
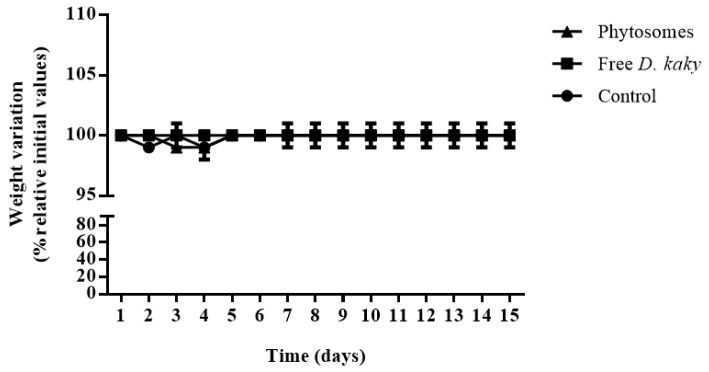
The variation in weight of rats during 15 days of oral administration of extract-loaded phytosomes (full triangles); free extract (full squares) and control (full circles) consisting of normal rats dosed with water. The data has been expressed by the means ± SD.

As observed in Figure 5, the oral administration of free *D. kaki* extract or loaded phytosomes did not influence the body weight during the 15-day period. No difference in other clinical signs was observed between the test group dosed with extract-loaded phytosomes and the free extract group. There was no mortality in either group.

#### 3.6.2. Evaluation of Changes in Organs Function

During the protocol, no changes were observed in this parameter. Normal rats showed an average value of glycaemia during protocol time around 156 ± 5 mg/dl. The group of free extract dosed had 158 ± 15 mg/dL and phytosomes dosed group exhibited 154 ± 22 mg/dL. All values corresponded to normal glycaemia [18]. 

The urine analysis is presented in Table 5. The values of the test group were similar to the control group except one case more of proteinuria, and one single case of ketonic bodies and bilirubin. In group dosed with free extract, animals presented more proteinuria and leucocytes. Glycosuria was not observed, and urine pH and density were similar for all groups.

The haematological parameters are summarized in Table 6. Animals from the test group showed similarity of haematological parameters with the control group. The observed values were similar as the reference for healthy Wistar rats [49,50,51].

Other parameters are summarized in Table 7. Some biochemical parameters of the test group were similar to control values such as creatinine, total proteins, low density lipoprotein (LDL) and high density lipoprotein (HDL). 

In this study, the ranges of total serum protein were 7.3 ± 0.02~7.33 ± 0.03 g/dL. This concentration was within the reference range of 6.3~8.6 g/dL for serum total protein concentration in Wistar rats [52].

The urea was slightly reduced in tested groups. According to referenced values [49], the renal function was accessed with the urea, creatinine parameters, and the oral administration of free extract, and phytosomes to the test groups did not change the renal function of these healthy rats.

The ALT level was reduced in both test groups when compared to the control group, and in the phytosome group, this reduction was greater than in the free extract. The reference values for ALT are generally between 17 and 50 U/L [50,51]. In the case of AST, the values were lower in the test groups. The AST was found to be more reduced in the group of rats administered with free extract than in those administered with encapsulated extract, but all values were within the referenced range [49]. Concerning serum ALP concentration, it was lower in the phytosomes group than in the other groups, but closer to the control group. In the free extract group, there was even an increase in relation to the control group. These biomarkers of liver function indicated that this function has not been altered in relation to normal function. However, there was a decrease in the expression of all of them.

Triglycerides and cholesterol were statistically different from the control. The free extract somehow reduced the LDL expression and increased the HDL expression. Gorinstein and colleagues already described the improvement of biochemical analysis during a 4 week diet supplemented with dry persimmon and with phenol-free dry persimmon [53,54]. In the systematic review and meta-analysis of randomized clinical trials evaluating the relationship between green tea catechin (GTC) and serum lipid levels including LDL, HDL, and triglycerides, it concluded that the consumption of GTCs was associated with a statistically significant reduction in levels of total and LDL cholesterol. However, there was no significant effect on HDL or triglyceride levels [55], in addition to the positive correlation of catechin with the free radical sequestration capacity, demonstrated in previous studies [56].

## 4. Discussion

Persimmon is a fruit that is known by its composition in nutritious and bioactive components, with a great potential for its use within the cosmetic and pharmaceutical industries [9,56,57,58,59,60,61,62]. This study and previous ones described persimmons as a fruit rich in PCs, such as gallic acid [63] and flavonoids [64]. 

The dosages of the total phenolic content in the persimmon fruit described in the literature varied widely. Denev and colleges determined 916.8mg GAE/100g FW [65]. In the studies of Veberic et al. with 11 different cultivars, values ranged from 12.7 to 29.5 mg GAE/100g FW [35]. The wide variability observed in these assays can be explained on the basis of the edaphoclimatic conditions, different opossum cultivars analysed, and stage of maturation, even when the fruit is more adequate for consumption. In addition, the different extraction methods applied and the analytical method protocols may have influenced the results [6]. Gallic acid was the most abundant PC in the extract and is well known in the traditional Chinese medicine, for its antioxidant properties, protecting from oxidative stress damages, increasing survival [34,66]. The phytosomes prepared in this work were able to encapsulate 99.3% of gallic acid present in the extract and 97.4% of total phenolic compounds.

This type of carrier system has been used when poor oral and/or erratic oral bioavailability of the polyphenols [67,68] occurred. In general, the encapsulation process can improve the rate of absorption, the extent of solubilisation in aqueous intestinal fluids and also to improve their ability to cross biomembranes [68,69]. The activity of these complexes has been widely described in several fields of application like cardiovascular [70], anti-inflammatory [71], hepatoprotective [72,73], anticancer [74], and even as cosmetics (anti-skin aging) [75].

The results showed that monodisperse phytosomes were successfully prepared with size ranges of less than 300 nm for both formulations over time at all T and RH conditions tested, given that this small particle size is important for oral absorption [76]. In this study, the size was managed regardless of the molar ratio, over 6 months of storage at different T and RH, which predicted the stability of the formulation. The size is an important factor for oral absorption and formulation stability in order to achieve a significant improvement in the bioavailability of persimmon PC. Previous studies have shown that a reduction in the size of the drug delivery systems improved their intestinal absorption [77,78]. It should also be noted that it has been described that size lower than 277 nm increased the emulsion stability of vitamin E-loaded phytosomes [79]. Moreover, some findings in lycopene nanoemulsions also showed that droplet sizes between 100 and 200 nm exhibited high anti-radical efficiency and antioxidant activities [80,81]. 

The narrow size distribution of carriers guarantees the uniformity and predictability of delivery of bioactive compounds [82]. The value of PI, indicative of the degree of heterogeneity regarding sample size, is another parameter possible to assess. There is an inherent polydispersity within any given batch of submicron particles, which needs to be controlled for predictable interaction with cells, otherwise different batches of the same material might display different results in cell studies [83]. The PI showed that monodisperse populations of phytosomes were obtained, especially for molar ratio of 1:1, at all temperatures. The higher ratio led to some agglomeration. A small value of PI (<0.3) indicated a homogenous population, while a larger PI (>0.3) meant a high heterogeneity [84,85]. 

Li et al. (2011) optimized the formulation of the extract containing self-emulsifying delivery systems, where droplet dispersion sizes ranged between 100–250 nm, having 44.48 mg total flavonoids/g persimmon leaf extract. The oral bioavailability of quercetin and kaempferol in vivo in beagle dogs was significantly enhanced by smaller nanoparticles, compared with commercial tablets. According to the in vitro-in vivo correlation study, the oral bioavailability enhancement was due to the increased drug concentration within the GI tract and its absorption area [86]. 

The phytosomes prepared in this work exhibited negative zeta potential, between −20 and −40 mV. In general, high positive or high negative zeta potential values may cause strong repulsive forces, whereas particles with similar electric charges may cause repulsion between them, preventing aggregation of the particles and easy re-dispersion [87,88]. In a scenario with combined electrostatic and steric stabilization, it is desirable that a minimum zeta potential of ± 20 mV [42], whereas a high zeta potential (positive or negative) should maintain a physically stable system [89]. However, it should be noted that negatively charged particles are cleared away more slowly from the blood when compared to positively charged particles, remaining within the bloodstream for longer periods of time [90]. This suggests the potential of this formulation was also related to the EE calculated, which was close to 100%.

In studies with nanoparticles, Shyam et al. [91] developed gallic acid phospholipids complex in different ratios to improve the lipophilic properties of gallic acid and to overcome its poor absorption because of less lipophilicity. They reported that gallic phospholipids complex was an effective scavenger of DPPH radicals with strong antioxidant activity [91]. Maiti et al. [92] reported that phytosomes of curcumin (flavonoid from turmeric, *Curcuma longa*) and naringenin (flavonoid from grape fruit, *Vitis vinifera*) showed higher antioxidant activity than pure curcumin in all dose levels tested [92], which is in accordance with present results, where pure quercetin alone showed an antioxidant activity lower than the free extract and phytosomes tested. Both tested formulations were very stable in terms of antioxidant activity. In 6 months of storage, at different conditions of T/RH, they exhibited losses of antioxidant activity of less than 20%, which did not occur for the free extract, or even for instance, for the positive control (quercetin).

Naik et al. [93] prepared grape seed phytosomes which were composed by oligomeric polyphenols (grape proanthocyanidins or procyanidins from grape seed extract, *Vitis vinifera*) complexed with phospholipids. They indicated that total antioxidant capacity and stimulation of physiological antioxidant defences increased through a network of mechanisms that extended beyond their great antioxidant potency, offering a marked protection for the cardiovascular system and other organs [93]. In the present study, it was observed that repeated-dose oral administration of phytosomes and extract in healthy rats did not change organ functions or body weight after a 15-day period. There were no mortalities or any deviation from normality in regards to clinical signs after the oral administration of the extract-loaded phytosomes or the free extract. All haematological, biochemical and urine parameters were within the reference values, suggesting the in vivo safety of this novel oral dosage form and the potential therapeutic use of this dosage form in order to deliver these active components of the fruit. The potential applications are varied, and given the antioxidant potential and already demonstrated anti-inflammatory effects [9], clinical situations associated to chronic inflammation might benefit from administration of the extract in phytosomes. Future studies should aim at characterising the pharmacokinetics of these phytosomes, with special interest in their bioacessibility and bioavailability.

## 5. Conclusions

This study showed, for the first time, phytosomes with aqueous persimmon extract for potential application as a food supplement. Monodisperse phytosomes were successfully prepared with size ranges of less than 300 nm. In contrast to the free extract, the extract-loaded phytosomes had higher antioxidant activity over 6 months and under accelerated storage conditions. These phytosomes were also able to encapsulate 97.4% of total phenolics, protecting them from the harsh environment like heat and humidity. From the PCs quantified in the persimmon extract, gallic acid was present in the highest concentration; the epicatechin was the second most abundant compound; followed by chlorogenic, *p*-coumaric, catechin, and ferulic acid; and finally, fisetin, protocatechuic acid, and caffeic acid. Additionally, the results suggested that the polyphenolic extracts of persimmon in phytosomal formulation were an interesting dosage form for nutraceutical purposes. To the best knowledge of the authors, there is no record of any formula with persimmon fruit extract involving phytosomes. With this study, the authors could suggest that these systems may be considered as promising candidates for the future delivery of bioactive agents of the persimmon fruit extracts. However, further integrated research is still needed to improve meticulousness, deepen the mechanisms of action, and its chronic use. 

## Figures and Tables

**Table 1:** The concentration of total PCs.

Sample	[Phenolic Compounds]	
	mg GAE/L	mg GAE/100g FW
Extract	9620 ± 779.4	641 ± 51.96
Added extract	1112 ± 34.6	-
Supernatant	70.9 ± 4.9	-

**Table 2:** Calibration curves for 9 PCs.

PC	R_t_ (min)	λ_max_ (nm)	Working Range (µg/mL)	Curve Equation	*R* ^2^	LOQ (µg/mL)	LOD (µg/mL)
Gallic acid	1.1	270	0.2–10	y = 112256x + 2292.6	0.9998	0.2	0.1
Protocatechuic acid	1.8	260	0.1–20	y = 143215x − 4179.8	0.9999	0.1	0.05
Chlorogenic acid	2.1	327	0.125–6.25	y = 110637 + 10485	0.9992	0.125	0.0625
Catechin	2.6	280	0.6–90	y = 28623x − 12629	0.9991	0.6	0.3
Caffeic acid	3.2	324	0.05–10	y = 212078x − 1682.3	0.9997	0.05	0.025
Epicatechin	3.5	280	0.5–25	y = 29894x − 1630.5	0.9998	0.5	0.125
*p*-coumaric acid	4.5	310	0.05–2.5	y = 260742x + 5928.8	0.9991	0.05	0.025
Ferulic acid	5.1	323	0.075–3.75	y = 224909x + 5856.4	0.9992	0.075	0.0375
Fisetin	7.5	360	0.2–2.5	y = 124764x − 12706	0.9991	0.2	0.1

**Table 3:** The concentration of the 9 PCs in the persimmon extract.

PC	Concentration (mg/100 g FW)
Gallic acid	2.794 ± 0.263
Protocatechuic acid	0.005 ± 0.00
Chlorogenic acid	0.171 ± 0.016
Catechin	0.071 ± 0.00
Caffeic acid	0.001 ± 0.00
Epicatechin	0.401 ± 0.045
*p*-coumaric acid	0.097 ± 0.004
Ferulic acid	0.027 ± 0.002
Fisetin	0.016 ± 0.00

**Table 4:** The characterization of the phytosomes.

Parameters		Phytosomes 1:1 (Molar Ratio)			Phytosomes 1:2 (Molar Ratio)		
		4 °C	25 °C	40 °C	4 °C	25 °C	40 °C
0 months	Average particle size ± SD (nm)	134.8 ± 36.0	177.1 ± 70.0	236.6 ± 65.6	92.8 ± 26.6	193.3 ± 92.2	153.7 ± 42.3
	Polydispersity Index (PI)	0.184	0.184	0.236	0.347	0.347	0.288
	Zeta (mV)	−41.74 ± 0.02	−40.15 ± 0.03	−27.62 ± 0.01	−40.07 ± 0.01	−41.31 ± 0.02	−27.62 ± 0.01
3 months	Average particle size ± SD (nm)	134.8 ± 36.0	177.1 ± 70.0	324.0 ± 146.2	92.8 ± 26.6	193.3 ± 92.2	226.6 ± 150.8
	Polydispersity Index (PI)	0.184	0.184	0.236	0.347	0.347	0.288
	Zeta (mV)	−41.74 ± 0.01	−40.15 ± 0.01	−27.62 ± 0.01	−40.15 ± 0.01	−41.31 ± 0.01	−30.19 ± 0.03
6 months	Average particle size ± SD (nm)	165.2 ± 57.6	174.4 ± 82.8	161.1 ± 62.5	228.0 ± 37.4	241.1 ± 129.4	183.5 ± 126.9
	Polydispersity Index (PI)	0.103	0.220	0.141 0.210	0.197	0.233	0.300
	Zeta (mV)	−20.07 ± 0.01	−22.10 ± 0.02	−21.03 ± 0.01	−20.07 ± 0.01	−21.03 ± 0.01	−20.02 ± 0.01

**Table 5:** Urine analysis.

Group	Ketonic bodies	Glucose (mg/dL)	Proteinuria	Density	pH	Nitrites	Blood	Bilirubin	Uribilinogen	Leucocytes
Control	-	-	1 positive case	1.00-1.02	5.0-7.5	-	-	-	N	1 positive case
Phytosomes	1 positive case	-	2 positive case	1.01-1.03	6.0-7.5	-	-	1 positive case	N-4	1 positive case
Free extract	-	-	4 positive cases	1.01-1.03	5.0-8.0	-	-	-	N-4	2 positive cases

- indicates result is not detected; N, within normal range.

**Table 6:** The haematological parameters after 15 days of oral administration. The data has been expressed by the means ± SD.

Haematological Parameters	Negative Control	Free Extract	Extract-Loaded Phytosomes
RBC (× 10^12^/L)	8.1 ± 0.2	7.8 ± 0.5	8.0 ± 0.4
Hb (g/dL)	15.4 ± 0.7	14.7 ± 0.7	15.0 ± 0.5
Haematocrit (%)	55.8 ± 1.2	53.1 ± 3.7	54.2 ± 3.0
WBC (10^9^/L)	3.3 ± 0.0	4.0 ± 0.6	5.0 ± 0.4
MO (%)	0.4 ± 0.1	0.7 ± 0.8	1.6 ± 1.2
NEU (%)	21.3 ± 2.1	19.0 ± 1.2	19.2 ± 4.4
EO (%)	1.7 ± 0.2	1.7 ± 1.8	1.6 ± 0.90
BAS (%)	0.0 ± 0.0	0.3 ± 0.2	0.2 ± 0.1
LYM (%)	76.7 ± 2.5	78.3 ± 3.6	77.4 ± 6.3
Platelets (10^9^/L)	541.0 ± 9.9	443.6 ± 112.3 ****	705.8 ± 123.3 ****
MCV (fL)	68.7 ± 0.1	67.70 ± 0.8	67.9 ± 0.8
MCH (pg)	19.0 ± 0.5	18.8 ± 0.4	18.8 ± 0.5
MCHC (g/dL)	27.6 ± 0.7	27.8 ± 0.8	27.7 ± 0.8

**** Statistically different from control with *p* ˂ 0.0001.

**Table 7:** The biochemical parameters after 15 days of oral administration. The data has been expressed as the means ± SD.

Biochemical Parameters	Control	Free Extract	Phytosomes
UR (mg/dL)	37.3 ± 2.08	35.8 ± 1.64	30.4 ± 1.95 *
CR (mg/dL)	0.48 ± 0.03	0.50 ± 0.01	0.46 ± 0.018
ALT (U/L)	42 ± 2.65	32.0 ± 2.12 ***	24.8 ± 2.39 ***
AST (U/L)	194.67 ± 5.86	86.4 ± 5.68 ***	107.4 ± 5.41 ***
ALP (U/L)	69 ± 3.61	90.2 ± 3.11 ***	63.8 ± 10.13 *
T-PROT (g/dL)	7.3 ± 0.02	7.32 ± 0.03	7.33 ± 0.03
CHOL (mg/dL)	52.67 ± 2.52	57.2 ± 1.48 *	57.4 ± 1.52 *
TRIG (mg/dL)	255.67 ± 4.04	273.0 ± 3.4687 ***	287.6 ± 2.77 ***
LDL (mg/dL)	2.00 ± 0.00	1.4 ± 0.55	2.2 ± 0.45
HDL (mg/dL)	43.67 ± 1.53	46.6 ± 1.14	42.6 ± 1.14

* Statistically different from control with *p* ˂ 0.05. *** Statistically different from control with *p* ˂ 0.0001.
